# Immunosurveillance of cancer cell stress

**DOI:** 10.15698/cst2019.09.198

**Published:** 2019-07-03

**Authors:** Seila Lorenzo-Herrero, Christian Sordo-Bahamonde, Segundo González, Alejandro López-Soto

**Affiliations:** 1 Departamento de Biología Funcional, Inmunología, Universidad de Oviedo, Oviedo, Spain.; 2 Instituto Universitario de Oncología del Principado de Asturias (IUOPA) Oviedo, Spain.; 3 Instituto de Investigación Sanitaria del Principado de Asturias (ISPA), Oviedo, Spain.

**Keywords:** cancer, cell stress, immune system, tumor immunity, immunosurveillance

## Abstract

Cancer development is tightly controlled by effector immune responses that recognize and eliminate malignantly transformed cells. Nonetheless, certain immune subsets, such as tumor-associated macrophages, have been described to promote tumor growth, unraveling a double-edge role of the immune system in cancer. Cell stress can modulate the crosstalk between immune cells and tumor cells, reshaping tumor immunogenicity and/or immune function and phenotype. Infiltrating immune cells are exposed to the challenging conditions typically present in the tumor microenvironment. In return, the myriad of signaling pathways activated in response to stress conditions may tip the balance toward stimulation of antitumor responses or immune-mediated tumor progression. Here, we explore how distinct situations of cellular stress influence innate and adaptive immunity and the consequent impact on cancer establishment and progression.

## CANCER AND THE IMMUNE SYSTEM: AN OVERVIEW

The immune system exerts a constant surveillance to protect the organism against foreign threats (e.g. infections) and damaged cells undergoing stress or malignant transformation. Two distinct branches of the immune system cooperate to accomplish this protective function: innate and adaptive immunity. Innate immunity is the first reacting to tissue homeostasis perturbations, being in charge of recruiting adaptive immune cells to the injured tissue during inflammation. Pre-malignant lesions show disrupted homeostasis, thus favoring immune infiltration and chronic inflammation. The diverse immune cell types present in the tumor niche can either impair or enhance tumor development and, consequently, the prognostic value of the tumoral immune contexture varies depending on the type of cancer [1].

The immune system is able to eradicate the majority of arising tumors or even control advanced ones via activation of effector responses. CD8^+^ T cells recognize specific tumor antigens presented via major histocompatibility complex class I (MHC-I) molecules, triggering the secretion of cytotoxic molecules and effector cytokines [e.g. interferon gamma (IFN-γ)] that result in the immune killing of the target cell. Nonetheless, persistent antigen stimulation leads to T cell exhaustion, a strategy commonly employed by tumor cells to evade antitumor responses [2]. Further, tumor cells promote T cell dysfunction through expression of ligands for inhibitory receptors, as, for instance, programmed cell death ligand 1 (CD274, best known as PD-L1). Anticancer therapies based on inhibitors of these immune checkpoints -commonly known as immune checkpoint blockade (ICB) therapies- are rendering encouraging clinical results, hence supporting the high efficacy of the immune system in tumor clearance [3]. Natural killer (NK) cells are innate lymphoid cells endowed with a strong antitumor cytotoxic function regulated by a complex array of activating and inhibitory surface receptors [4]. The expression of certain NK cell activating ligands is generally upregulated in malignantly transformed cells, thereby providing a strong stimulatory signal to NK cells that leads to tumor eradication during tumorigenesis and metastasis [5, 6].

Pro-inflammatory immune cells are also present in pre-malignant lesions and tumor niches, where they contribute to tumor establishment and growth. Tumor-associated macrophages (TAMs) comprise the majority of the innate immune infiltrate in solid tumors and predominantly display an alternatively activated M2-like phenotype, a differentiation state associated with poor prognosis in patients with cancer [7, 8]. Polarization of TAMs to an M1-like phenotype is normally correlated with better outcome, a fact that is prompting the development of anticancer strategies that induce macrophage reprogramming [9–11]. Unlike NK cells, chronic activation of TAMs and other innate immune cells, such as myeloid-derived suppressor cells (MDSCs), can potentiate tumor formation and development through modulation of physiological processes such as extracellular matrix and vascular remodeling or suppression of antitumor responses [8]. Suppression of effector immune responses is further achieved by recruitment of regulatory T (Treg) cells to the tumor site [12]. Concomitantly, mouse models with attenuated innate immune cell functions exhibited restricted tumor growth and invasion [13]. Pro-inflammatory cytokines released by these innate immune cells, such as TNF-α, further contribute to establish a pro-tumoral niche and support neoplastic cell proliferation and survival. Of note, immune evasion, together with inflammation, are two emerging hallmarks of cancer [14], underscoring the interplay of the immune system and cancer progression.

Here, we review different situations of cellular stress that modulate the interaction of the innate and adaptive immune system with cancer and how they tip the scale towards an immunosuppressive or antitumor state within the tumor.

## CELLULAR STRESS, IMMUNITY AND CANCER

Dysregulation of cellular homeostasis and chronic stress conditions can lead to malignant transformation. Adaptation to stress is mediated by a series of intrinsic signaling pathways that carve the immunogenic profile of the tumor cell and define their abilities to evade the immune system. In addition, the challenging conditions of the tumor microenvironment (TME) trigger cellular stress responses that both shape the tumor cell phenotype and regulate the functions of infiltrating immune cells. Altogether, stress conditions in the context of cancer play a central role in the interplay between immune cell subsets and tumor cells. Stress signaling pathways are highly intertwined with each other and can shift the balance towards tumor establishment and development or antitumor immunity. Herein, we provide an integrated view of distinct types of cellular stress and their impact on cancer immunosurveillance.

### Proteotoxic stress

Body temperature is tightly controlled by a thermoregulatory system in homeothermic animals, such as mammals. Temperatures above the physiological range generate a situation of thermal stress inside the cell that, at a molecular level, leads to a disruption of protein homeostasis and translates into a heat shock protein (HSP) response. Even though this response was first described in studies analyzing the effects of heat and it is considered a hallmark of thermal stress, proteotoxic stress conditions can be triggered by a myriad of stresses, including oxidative damage or genomic instability [15]. HSP response is mediated by activation of heat shock transcription factor 1 (HSF1) [16], which orchestrates a gene expression program involved in the adaptation to stress. HSF1 overexpression has been described in different types of tumors [17–19] frequently correlating with poor prognosis [20–22]. Thus, HSF1 has been shown to contribute to malignant transformation and tumor cell survival in breast cancer [23] and multiple myeloma (MM) [18], supporting earlier findings in models of spontaneous malignancy [24, 25]. In addition, a study in breast cancer cell lines unraveled that tumors carrying gain-of-function p53 mutations show survival advantage against proteotoxic stress due to HSF1 stabilization and activation [26]. Recent work further described an interaction between HSP response and oncogene activation in T cell acute lymphoblastic leukemia (T-ALL), given that NOTCH1 [Notch homolog 1, translocation-associated (Drosophila)] signaling induced HSF1 and ablation of this transcription factor eradicated tumor growth in NOTCH1-induced T-ALL models [19].

Heat stress and hyperthermia (fever-range temperatures) also have a direct effect on immunity, activating both the innate and adaptive components of the immune system [27]. In particular, heat stress has been shown to promote neutrophil recruitment into the TME in colon carcinoma-bearing mice exposed to whole body hyperthermia, which contributed to tumor rejection [28]. Likewise, HSF1 activation triggered by mild thermal stress leads to rearrangement of natural killer group 2, member D (CD314, best known as NKG2D) receptor molecules in clusters along the NK cell plasma membrane, resulting in enhanced *in vitro* antitumor NK cell activity against colon carcinoma cells [29]. Similarly, NKG2D ligand (NKG2DL) expression was markedly upregulated upon exposure to high temperature on the surface of a panel of tumor cell lines [29, 30]. Thus, HSF1 factor stimulates *MICA* (MHC class I polypeptide-related sequence) transcription under heat stress through binding to heat shock elements (HSE) in the promoter region of this NKG2DL [31], whereas inhibition [32] or silencing [33] of HSF1 abrogated the heat-induced upregulation of MICA in tumor cell lines. Moreover, treatment of MM cell lines with HSP90 inhibitors induces MICA surface expression [34], further supporting the importance of HSF1, since HSP90 has been shown to sequester HSF1 in unstressed cells, thereby limiting its transcriptional activity [35]. Interestingly, HSPs can also stimulate antitumor adaptive immunity by promoting antigen presentation of tumor-related peptides [36–38]. Particularly, enhanced dendritic cell (DC) and T cell infiltration owing to HSP70-dependent tumor chemokine production has been reported in mice challenged with Lewis lung carcinoma (LLC) cells [39] and in Eμ-Myc mouse lymphoma models [40]. Additionally, HSP70 present on the surface of tumor-released exosomes improved *in vitro* NK cell cytotoxicity against pancreas and colon carcinoma [41] and lymphoma [42] cell lines.

**Figure 1:**
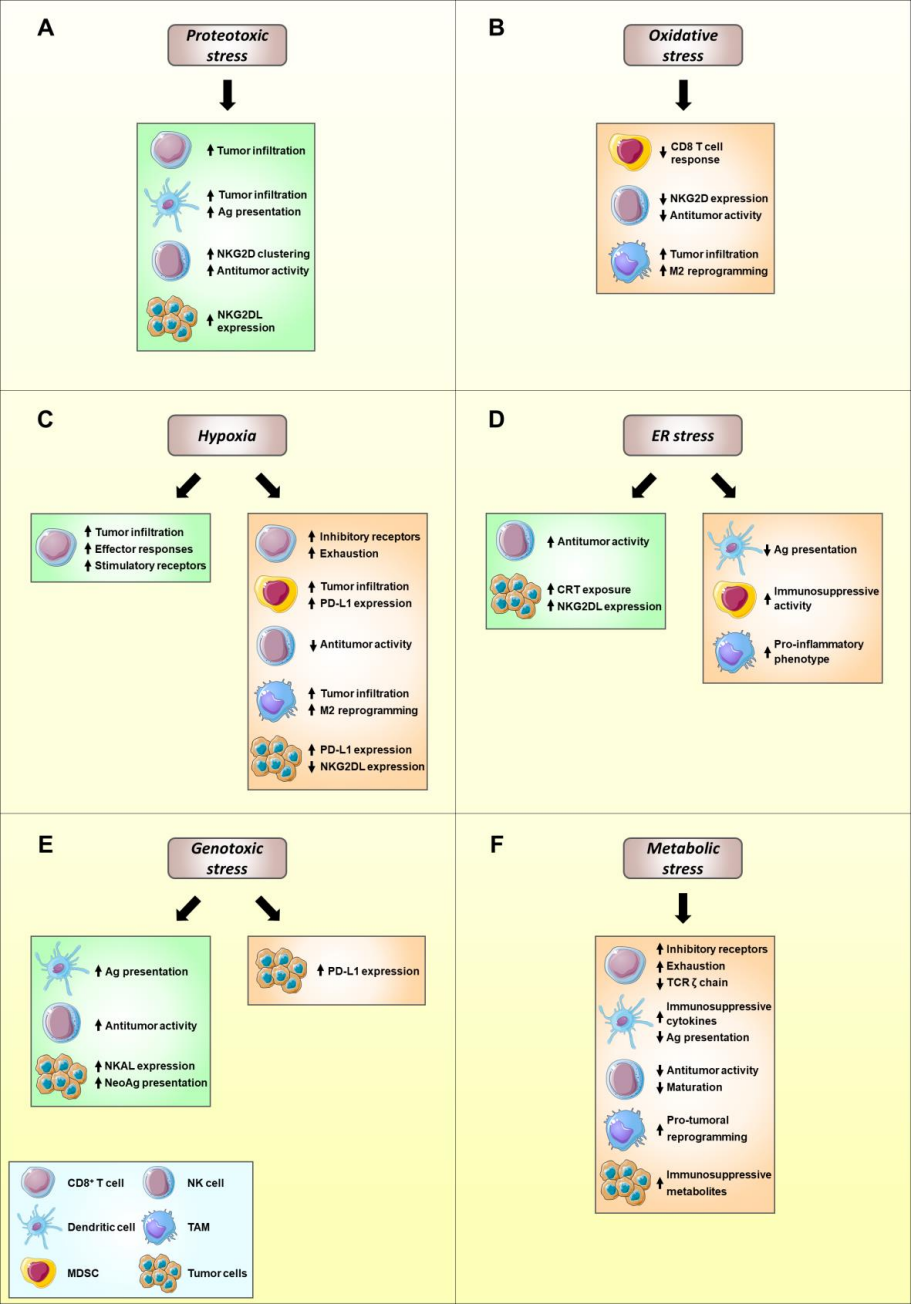
FIGURE 1: Modulation of immune function and tumor immunosurveillance by cell stress. The distinct signaling pathways implicated in the adaptation to cell stress can positively modulate antitumor immune responses (green), thus favoring tumor elimination, or can be detrimental to cancer immunosurveillance (orange), promoting tumor development and growth. The balance between both effects ultimately defines the crosstalk between the immune system and the tumor. Proteotoxic **(A)** and genotoxic **(E)** stress predominantly stimulate effector immune subsets, potentiating the recognition and killing of tumor cells. Oxidative **(B)** and metabolic **(F)** stress mainly impair effector immune cell functions and promote the functions of protumoral immune subsets, such as MDSCs, therefore favoring tumor progression. Hypoxia **(C)** and ER stress **(D)** exert a double-edge role in cancer immunosurveillance. Ag, antigen; CRT, calreticulin; MDSC, myeloid-derived suppressor cell; NKG2DL, NKG2D ligand; TAM, tumor-associated macrophage; TME, tumor microenvironment. Illustrations adapted from Servier Medical Art (http://www.servier.fr/servier-medicalart).

Collectively, these studies support that proteotoxic stress, and activation of the HSP response through HSF1, ultimately favors the antitumor immune response **(Figure 1A)**.

### Oxidative stress

Cellular aerobic metabolism renders oxygen-derived byproducts, commonly known as reactive oxygen species (ROS). As ROS are highly reactive chemicals that can damage different biomolecules within the cell, including DNA [43], cells rely on antioxidant enzymatic systems to balance their redox potential. Dysregulation of such balance and increased endogenous ROS levels lead to oxidative stress, which, if not resolved, can cause cell death [43, 44]. ROS function as secondary messengers that activate transcription factors implicated in cell adaptation to stress and regulation of immunity (e.g. forkhead box, class O (FoxO) [45] and nuclear factor Kappa B (NF-κB) [46]) and modify the enzymatic activity of redox-sensitive proteins [47]. Consistent with these, several studies have documented a connection between cell proliferation and ROS, since these metabolites, mostly hydrogen peroxide (H_2_O_2_), can inactivate phosphatases that negatively regulate proliferative pathways [48–50]. Along with increased proliferation, exacerbated ROS induce DNA damage, thereby favoring tumorigenesis [51] (see *Genotoxic stress*). Additionally, ROS are implicated in tumor angiogenesis [52], invasion and metastasis [53] as well as evasion of apoptosis [54].

Noteworthy, the oxidative TME influences the immune system surrounding the tumor. A recent study revealed that oxidative stress-induced Treg cell apoptosis results in release of ATP, which is subsequently converted to immunosuppressive adenosine (see *Metabolic stress*), hence contributing to tumor development and resistance to ICBs [55]. In lung and breast cancer models, TAMs required ROS to infiltrate the tumor niche and differentiate into a pro-tumorigenic M2 phenotype [56]. MDSCs isolated from patients with head and neck cancer as well as from tumor-bearing mice showed higher ROS content, which was ascribed to upregulation of NADPH oxidase 2 (NOX2) [57]. Interestingly, lack of NOX2 activity in this model negatively affected the ability of MDSCs to limit antigen-specific CD8^+^ T cell activation. Direct inhibition of ROS rendered similar results in fibrosarcoma-bearing mice [58], suggesting that tumor-related MDSCs contribute to immunosuppression through ROS production. *Ex vivo* studies in metastatic renal cell carcinoma further showed that co-culture of suppressed T cells and MDSCs in the presence of the H_2_O_2_ scavenger catalase mostly restores IFN-γ production in T cells to physiological levels [59]. Myeloid NOX2-deficient mice showed reduced melanoma metastasis and enhanced IFN-γ production in NK cells, whereas NK cell depletion reestablished metastatic potential, implying that the cancer malignancy control exerted by NK cells is hampered by myeloid-derived ROS [60]. In line with this, phagocytes, via ROS production, efficiently downregulated NKG2D and NKp46 (natural cytotoxicity receptor 1; also known as NCR1 or CD335) surface expression *in vitro*, which has been proposed to mediate NK cell deficiency in patients with acute myeloid leukemia [61]. Concomitantly, NK cell dysfunction in chronic myelogenous leukemia (CML) is likely to be caused by tumor-produced ROS, since NK cell cytotoxic capacity against primary tumor cells obtained from patients affected with this malignancy was restored in the presence of catalase [62]. In contrast, upregulation of *MICA* and *MICB* (MHC class I polypeptide-related sequence B) gene expression has been reported in CaCo-2 colon carcinoma cell line upon oxidative stress [63], an effect that could strengthen NK cell recognition and tumor cell elimination.

Taken together, these data bring to light a double-edged role of ROS in the antitumor immune response, emphasizing its link to cancer immunosuppression **(Figure 1B)**.

### Hypoxia

Molecular oxygen is crucial for cellular metabolism in aerobic organisms and its deprivation, referred to as hypoxia, leads to cell stress. The microenvironment of solid tumors typically displays low-oxygen conditions. Under hypoxia, a series of oxygen-sensing mechanisms including, but not limited to, the unfolded protein response (UPR) and mTOR (mammalian target of rapamycin) signaling [64] trigger an adaptive transcriptional response largely mediated by hypoxia-inducible factors (HIF). HIF transcription factors are heterodimer proteins comprised by an oxygen-sensitive α-subunit that, upon activation, translocates into the nucleus and joins the stable β-subunit. This complex binds to HIF-responsive genes through hypoxia response elements (HRE) and modulate glucose and lipid metabolism and redox homeostasis in hypoxic cells [65]. HIF-responsive genes participate in essential aspects of tumor development such as metabolism [66, 67], angiogenesis [68, 69], proliferation [70] or invasion and migration [71–73]. Consequently, HIF-dependent signaling, which is highly activated in hypoxic tumor cells, strongly contributes to tumor growth and progression.

In addition to its promoting role in tumorigenesis, hypoxia also impinges on cancer progression through the modulation of immunity. Hypoxia has been shown to upregulate PD-L1 expression in a battery of mouse and human tumor cell lines [74, 75]. Accordingly, silencing of endothelial PAS domain protein 1 (*EPAS1*; best known as *HIF2A*) in clear cell renal cell carcinoma [75] or *HIF1A* in melanoma and prostate cells [76] reduced PD-L1 expression and restored cytotoxic T lymphocyte (CTL)-mediated tumor cell killing *in vitro*. Germline mutations in genes that govern the Krebs cycle, such as succinate dehydrogenases, lead to induction of HIF1 subunits in paraganglioma [77–79], which could explain the enhanced expression of PD-L1 found in this type of cancer [80]. Likewise, hypoxia upregulates PD-L1 expression in tumor-promoting immune cells, mainly MDSCs [74], further favoring immune tolerance. This upregulation may be mediated by pyruvate kinase M2 (PKM2), as it binds to HRE on the *CD274* promoter together with HIF1α [81]. Hypoxic CD8^+^ T cells show elevated levels of inhibitory receptors, including programmed cell death 1 (PDCD1; best known as PD-1) and lymphocyte activating gene 3 (CD223; best known as LAG3) [82], thereby contributing to T cell exhaustion. Nonetheless, under hypoxia, CD8^+^ T cells also upregulate stimulatory molecules, as TNF receptor superfamily member 18 (TNFRSF18, commonly referred to as GITR) [82].

Tumor-promoting immune cells are recruited to hypoxic regions in solid tumors owing to the secretion of chemotactic factors, such as colony stimulating factor 1 (CSF1) or vascular endothelial growth factor (VEGF), by hypoxic tumor cells. In glioblastoma-challenged rats, hypoxic areas displayed a higher number of infiltrating TAMs, where they were re-educated towards an immunosuppressive M2-like phenotype [83]. The hypoxic TME of mice bearing LLC tumors fine-tunes the functional profile of infiltrating M2-like macrophages, leading to upregulation of HIF-dependent genes such as *VEGFA* [84]. Along similar lines, TAMs exhibit high levels of both HIF-α isoforms in response to hypoxia and, in consonance, myeloid-specific deletion of HIF1α [85] or HIF2α [86] has recently been correlated to reduced tumor growth and better outcome in breast cancer and colon carcinoma, respectively. In addition, HIF1α activates a pro-angiogenic program in macrophages that promotes vascular remodeling neo-angiogenesis within the TME. Hypoxic TAMs upregulate the negative regulator of mTOR REDD1 (regulated in development and DNA damage responses 1), enhancing abnormal tumor vessel growth (non-productive angiogenesis) and metastasis [87]. MDSCs preferentially infiltrate hypoxic regions driven by hypoxia-inducible tumor-derived factors, being C-C motif chemokine ligand 26 (CCL26) a clear example in hepatocellular carcinoma-associated MDSCs [88]. Ectonucleoside triphosphate diphosphohydrolase 2 (ENTPD2), an ecto-ATPase key for MDSC function and accumulation, is highly expressed in hepatocellular carcinoma cell lines owing to tumor hypoxia [89]. Further, tumor-associated hypoxia enhances Treg cell infiltration and accumulation via chemotactic factors, such as C-C motif chemokine ligand 28 (CCL28), promoting tumor tolerance and angiogenesis in ovarian cancer [90]. VEGF also plays an important part in Treg cell infiltration, since deficiency of neuropilin-1 (NP-1), a factor that responds to VEGF, impaired Treg cell infiltration and decreased tumor growth in spontaneous melanoma models [91].

Hypoxia has been shown to stimulate infiltration and antitumor effector function of CD8^+^ T cells in mouse models of implanted tumors [82, 92, 93]. Contrarily, experimental evidence suggests a negative role of hypoxia in the crosstalk between NK cells and tumor cells. Hypoxic conditions downregulated MICA on the surface of tumor cells by different mechanisms, including shedding of this NKG2DL mediated by HIF1α-dependent upregulation of the matrix metalloproteinase A disintegrin and metalloproteinase domain-containing protein 10 (ADAM10) [94, 95]. Further, NK cell cytotoxic killing of breast cancer cells is diminished by hypoxia through secreted factors that impair NK cell development [96] and tumor cell activation of autophagy that leads to degradation of granzyme B (GZMB) in autophagosomes [97]. Similarly, microvesicles produced by hypoxic lung carcinoma and CML cells attenuated NK cell antitumor responses *in vitro*, presumably via transforming growth factor beta 1 (TGF-β1) and miR23a [98]. Surprisingly, NK cell adaptation to hypoxia could support tumor progression in lung and colon carcinoma through enhancement of angiogenesis [99].

Collectively, while stimulating CD8^+^ T cell antitumor activity, the hypoxic TME hinders NK cell function, thus illustrating the enormous complexity of the role of hypoxia in modulating cancer immunosurveillance **(Figure 1C)**.

### Endoplasmic reticulum stress

Cells undergo endoplasmic reticulum (ER) stress under conditions that compromise the protein folding machinery or produce uncontrolled protein synthesis and load in the lumen of the ER. Under these circumstances, an adaptive homeostatic response, the UPR, is activated within the cell in order to restore ER proteostasis or induce apoptosis depending on the strength of the stress signal. As already mentioned, disruption of protein synthesis activates the HSP response as well (see *Proteotoxic stress*). Three ER stress sensors are in charge of initiating the UPR signaling cascade: the inositol-requiring enzyme-1α (IRE1), the protein kinase RNA-like ER kinase (PERK) and the activating transcription factor 6 (ATF6) [100].

Stress conditions typically related to the TME, such as hypoxia or oxidative stress, lead to an imbalance in proteostasis that triggers ER stress responses. Activation of UPR mediators in cancer allows not only adaptation to the microenvironment, but also tumor cell invasion and therapy resistance [101, 102]. Notwithstanding, extended ER stress can be detrimental to tumor progression through activation of apoptosis [103]. Prior to apoptosis, ER stress leads to surface presentation of danger-associated molecular patterns (DAMPs), such as the ER chaperone calreticulin (CRT), which elicits pro-inflammatory responses and immunogenic cell death (ICD – reviewed in [104]). In this line, drug-induced hyperploid colon carcinoma cells, which display constitutive ER stress, showed increased CRT surface exposure *in vitro*, which correlated with reduced tumor formation *in vivo* [105, 106]. Likewise, human hyperploid cell lines have been shown to upregulate surface expression of NK cell activating ligands, rendering these cells more susceptible to NK cell-mediated killing *in vitro* [107]. Collectively, these studies outline the relevance of the antitumor effect exerted by sustained ER stress in tumor immunosurveillance of cells with deviant karyotypes [108].

Activation of ER stress also constitutes an extensively described feature of tumor-infiltrating immune cells. Profiling studies in ovarian cancer-infiltrating DCs in human and mouse specimens revealed marked upregulation of ER stress effectors, especially corresponding to the IRE1 arm [109]. Conditional deletion of X-box binding protein 1 (*XBP1*), the main target of IRE1, in DCs [109] or in CD4^+^ T helper cells [110] compromised tumor progression in orthotopic murine models while supported T cell proliferation and function at tumor sites, defining a pro-tumoral role for IRE1 through impairment of cancer immunosurveillance. Noteworthy, compared to their healthy counterparts, tumor infiltrating MDSCs exhibit higher levels of DNA damage inducible transcript 3 (DDIT3; also known as CHOP), a proapoptotic transcription factor downstream of PERK branch that is key for MDSC turnover and immunosuppressive function [111, 112]. Noteworthy, ER-stressed tumor cells have been recently shown to produce yet-unknown soluble factors that drive UPR activation in immune populations in a process called transmissible ER stress. Conditioned media from murine tumor cells undergoing ER stress induced upregulation of UPR markers and promoted a pro-inflammatory state in macrophages [113] and downregulated cross-presentation to CD8^+^ T cells in myeloid DCs [114]. Whether this phenomenon also affects other immune subsets remains to be determined.

Despite the extensive studies performed concerning ER stress in tumors, the role of ER stress in the crosstalk between tumor cells and effector immune populations, such as NK cells and cytotoxic T cells, remains to be fully elucidated **(Figure 1D)**.

### Genotoxic stress

The integrity and stability of the genome is critical for cell survival. Nonetheless, DNA is continually exposed to intrinsic and extrinsic factors that cause lesions and threaten cell homeostasis. Since DNA anomalies are a daily constant, cells have developed a complex system to sense the damage coupled to specific repair mechanisms, such as base excision repair (BER) or homology-directed repair (HDR), to restore DNA integrity. Activation of the DNA damage response (DDR) arrests the cell cycle to cope with the lesions and, if the repair is unsuccessful, trigger apoptotic cell death [115].

Cells with a defective or overwhelmed DNA repair machinery count with high mutation rates and the resultant genetic instability can lead to malignant transformation [116]. In consonance, pre-cancerous lesions and early arising tumors display an accumulation of DNA damage and an activated DDR network, a hallmark of carcinogenesis [14, 117–119]. This mutational signature contributes to clonal expansion and cancer progression and intervenes in aspects as distinct as the response to therapy [120] or the interaction with the immune system. For instance, genomic instability leads to NF-κB and interferon regulatory factors (IRFs) activation, resulting in a pro-inflammatory and pro-survival state in tumor cells [121], features generally linked to immune evasion.

Immunosurveillance of DNA damage entails diverse strategies that allow immune detection and elimination of cells undergoing genotoxic stress, including tumor cells. Tumor-associated genome instability can favor the generation of mutant peptides with novel epitopes, known as neoantigens, which are presented by MHC molecules on the cell surface, resulting in T cell-mediated antitumor cytotoxic responses. A significant positive association between overall rate of mutation, predicted neoepitope load and immune cytolytic activity has been described in a wide variety of human cancers [122]. In line with this, studies analyzing the response rates of patients with cancer to ICB, such as anti-CTLA-4 in melanoma [123] or anti-PD-1 in non-small cell lung cancer (NSCLC) [124], observed a positive correlation between the mutational burden and the efficacy of the therapy. On the basis of these findings, neoantigens arise as an important part of immunosurveillance in cancer, as tumors are characterized by persistent genotoxic stress and higher mutation load than healthy tissues.

NK cells are central in the immunosurveillance of cells suffering DNA damage. Genotoxic agents induced the expression of NKG2DLs in healthy and tumor cells from human and mouse origin, thereby promoting NK cell activation and lysis of the affected cells [125]. This upregulation was essentially mediated by ataxia-telangiectasia mutated (ATM)- and Rad3-related (ATR) protein kinases, which act as DNA sensors during DDR. Furthermore, NK cell antitumor function is stimulated upon DNA damage induction in MM cells promoting an upregulation of poliovirus receptor (PVR), a ligand for the NK cell activating receptor CD226 (best known as DNAM-1) [126]. In contrast with this immunostimulatory role of genotoxic stress, recent research shows that expression of the inhibitory immune checkpoint PD-L1 is increased in cancer cells by the DNA double-strand break (DSB) pathway in an ATM/ATR-dependent fashion [127].

ATM is responsible for p53 activation and stabilization in the context of DNA damage, a protein also implicated in regulating NK cell ligand expression [128]. Pharmacological induction of genotoxic stress boosted surface expression of ULBP1 and ULBP2 [UL16 binding protein 1/2] in a p53-null NSCLC cell line bearing wild-type p53 but not in its mutant counterpart [129]. Similar results were obtained upon pharmacological reactivation of p53 in human tumor cell lines [130]. Moreover, NK cell activating ligand expression is also upregulated in MM cell lines owing to ROS-dependent activation of the DDR pathway [131]. Taken together, these studies bring to light the DDR as a master regulatory pathway of NK cell ligand expression and NK cell-mediated cancer immunosurveillance.

Another regulator of the immune response to healthy and tumor cells undergoing genotoxic stress is transmembrane protein 173 (TMEM173, best known as STING). The STING pathway is in charge of detecting cytoplasmic DNA and it has been linked to inflammatory responses via activation of IRF3 and NF-κB [132]. Its significance in antitumor immunity was brought to attention by *in vivo* experiments where mice deficient in STING [133] or cGAS (cyclic GMP-AMP synthase) [134], an upstream component of the pathway, failed to reject tumor growth. Treatment with STING agonists was shown to enhance antitumor immunity in diverse tumor mouse models [135, 136] and cooperated with ICB therapy in tumor removal in prostate cancer-bearing mice [137]. Further, STING signaling pathway is crucial in DC priming and effective cross-presentation to CD8^+^ T cells. Infiltrating DCs phagocyte tumor cells and remaining cytosolic DNA likely triggers STING activation, promoting IFN-dependent priming of immune responses [133, 138]. Coupled to these observations, it was demonstrated that induction of mouse NKG2DLs ensued by DDR relies on a STING-dependent pathway [139].

Collectively, these findings suggest a role for the DDR in activating antitumor immune responses mediated by effector immune populations, mainly NK cells **(Figure 1E)**.

### Metabolic stress

Multicellular organisms normally count with a steady supply of nutrients and their individual cells control nutrient uptake through growth factor signals. Excessive or insufficient growth factor-regulated nutrient uptake can negatively affect the metabolic machinery leading to cell stress. When nutrients are scarce, inhibition of macromolecule biosynthesis together with autophagy activation are the major strategies that cells employ to adapt. On the other end, cells experience nutrient excess when ROS levels exceed normal values [140], that is, in conditions of oxidative stress, an issue already discussed in this review (see *Oxidative stress*). Yet, rapidly dividing cells need to increase their nutrient uptake to fulfill their bioenergetics demands, such is the case of tumor cells, which have developed an altered metabolism towards anabolic pathways to sustain proliferation and counteract the nutritional stress associated to tumorigenesis [141]. This reprogrammed metabolism has been documented in many types of tumors and is nowadays considered a hallmark of cancer [14, 142]. A gene-profiling study analyzing tumor metabolic signatures discovered a great mutation rate affecting the whole network of metabolic pathways, although the distribution of genetic alterations differed between tumors [143]. These data further reinforce the metabolic dysregulation associated to cancer. Of note, metabolic reprogramming in tumor cells can be controlled by different stress pathways already issued in this review, mainly hypoxia and oxidative stress, as oxygen and ROS are integral elements of normal cellular metabolism [66, 144, 145]. Tumor cells display a remarkably increased glucose consumption compared to healthy cells, resulting in higher glycolytic flux and production of ATP and lactate, a process referred to as the Warburg effect [142]. This glycolytic switch has been associated to oncogene activation (e.g. Myc, Ras) and mutation of tumor suppressors (e.g. p53) [146, 147]. Tumor cells can also scavenge other biomolecules, such as lipids and amino acids, from the extracellular space [148]. Collectively, this abnormal tumor nutrient consumption derives in a depletion of the available molecules, which creates a nutrient-poor TME. The tumor addiction to distinct metabolic pathways opens a window for antitumor therapies that disrupt cancer metabolism. Supporting this notion, approaches targeting components of the bioenergetic metabolism, such as glucose transporter 1 (GLUT1) inhibitors, show promising antineoplastic results in preclinical studies [147].

The protective function and homeostasis of immune cells are also controlled at a metabolic level. As an illustration, CD4^+^ and CD8^+^ T cells experience a characteristic metabolic reprogramming that shape their maturation and activation upon antigen stimulation [149, 150]. In consonance, cancer progression causes metabolic alterations in tumor-associated immune cells, thereby impinging on cancer immunosurveillance [151]. Ovarian cancer-induced IRE1-XBP1-dependent ER stress results in impaired glucose import and metabolism in CD4^+^ T cells, thereby allowing tumor progression [110]. As a consequence of the altered tumor metabolism, the TME displays high levels of certain immunosuppressive metabolites, such as adenosine. Upon interaction of adenosine with adenosine A2a receptor (ADORA2A, also known as A2AR) results in diverse immunosubversive mechanisms, including, but not limited to: i) impaired DC activation and CD8^+^ T cell priming [152, 153]; ii) hampered NK cell maturation [154] and cytotoxic activity [155]; iii) increased production of immunosuppressive cytokines, such as IL-6 or IL-10 [152, 153]; and iv) upregulation of inhibitory immune checkpoints CTLA-4 and PD-1 [156–158]. Consequently, blockade of the adenosine signaling pathway has arisen as a new therapeutic approach in cancer, achieving encouraging results in combination with ICB therapy in preclinical models [159, 160] and improving T cell-mediated antitumor immune responses [160, 161]. Of note, adenosine levels in the TME are increased owing to enhanced activity of ectonucleoside triphosphate diphosphohydrolase 1 (ENTPD1, also known as CD39) and ecto-5'-nucleotidase (NT5E; best known as CD73) enzymes in response to hypoxia, once more highlighting the intense crosstalk between different types of cell stress. Furthermore, HIF signaling favors glycolysis over oxidative phosphorylation in hypoxic TAMs via upregulation of glycolytic genes, a metabolic adaptation that has been linked to a pro-tumoral M1-like phenotype of TAMs [151]. Conversely, lactic acid accumulation in the TME, a direct consequence of tumor growth, decreases the glycolytic flux [162] and fine-tunes TAMs towards an M2 phenotype [163], potentiating their immunosuppressive properties. Hence, glucose metabolism in TAMs is likely to be subjected to dynamic modifications during cancer progression that might adjust their phenotype to tumor requirements.

Other metabolic pathways, such as lipid and amino acid metabolism, are modulated in tumor infiltrating immune cells as well [164]. In addition to its well-established role as immune checkpoint, PD-1 receptor engagement has recently been shown to increase T cell fatty acid β-oxidation (FAO) during lipolysis, which was postulated to underpin PD-1-regulated longevity of T cells in the context of chronic infections and cancer [165]. Besides, M2-like TAMs in lung and melanoma TMEs express higher levels of glutamine metabolism enzymes, such as glutamate-ammonia ligase (GLUL) [163], a recently uncovered mediator of the immunosuppressive and pro-metastatic properties of TAMs [166]. Similar expression profiles were detected in TAMs isolated from human glioblastoma biopsies [167]. Further, macrophage-specific suppression of heme oxygenase 1 (HMOX1), an iron-releasing enzyme, correlated with reduced tumor growth in breast carcinoma [168] and prostate cancer [169] models, an effect attributed to activation of an M1 profile in TAMs. Therefore, metabolic reprogramming appears to be crucial in TAMs, since it mediates their pro-tumoral activities. DC dysfunction in tumors has been linked to metabolic changes as well. Interaction of SOCS3 (suppressor of cytokine signaling 3) with PKM2, and the consequent inhibition of the latter, altered DC metabolism and disrupted antigen presentation, resulting in attenuated T cell infiltration and augmented tumor growth in LLC-challenged mice [170]. Under ER stress conditions, DC inability to activate antitumor T cell responses is associated to dysregulation of the triglyceride biosynthetic pathway [109], supporting an active crosstalk between metabolism and cell stress responses. Concomitantly, effector immune cells rely on their metabolic plasticity to exert their function. Upon activation, NK cells suffer a dramatic increase in glucose uptake and a shift to glycolytic metabolism that are linked to IFN-γ production and GZMB expression [171–173]. A study in spontaneous lung cancer models revealed that NK cells acquire a dysfunctional state during tumor progression, characterized by attenuated cytotoxicity and altered cytokine profile, a phenotype ascribed to inhibition of glycolysis through aberrant expression of fructose-1,6-bisphosphatase (FBP1) [174]. Hence, glucose metabolism stands out as a critical player in the antitumor capacity of NK cells.

The extracellular amino acid reservoir is directly related to the function of different immune subsets as well [175]. At this respect, enhanced expression of arginase 1 (ARG1) and indoleamine 2,3-dioxygenase 1 (IDO1), enzymes that catalyze the degradation of arginine and tryptophan, respectively, have been documented in tumor infiltrating DCs [176–178] and MDSCs [179–182]. TAMs are also able to scavenge arginine from the TME to synthesize nitric oxide via ARG1 activity [164]. The absence of these amino acids results in downregulation of the TCR ζ chain in T cells [154, 183–185], limiting antigen-mediated activation and impairing their cytotoxic activity. Further, arginine starvation negatively affects T cell survival, cytokine production and proliferation [186, 187], eventually leading to T cell dysfunction. NK cells could also be susceptible to amino acid depletion, since low arginine concentrations reduced the expression of activating receptors and IFN-γ production in NK-92 cell line and inhibited the cytotoxic activity of isolated human NK cells [188]. Interestingly, tryptophan catabolism renders kynurenine, which enhances Treg cell generation [142, 154]. Considering the crucial role of amino acid availability in the TME, IDO-selective therapeutic strategies have been extensively studied [189, 190], although clinical trials did not progress as expected, arising doubts about amino acid metabolism targeting in cancer.

Altogether, these findings provide wide evidence for a crucial role of metabolic reprogramming in cancer progression and immune function **(Figure 1F)**.

## CONCLUDING REMARKS

As illustrated in this review, there is an extraordinarily intricate relationship between cell stress and cancer immunosurveillance. Indeed, each type of stress commonly results in diverse and opposite effects on the antitumor immunity, which may constitute a pitfall for harnessing drugs that trigger cell stress for the management of patients with cancer. For instance, while DNA damaging agents, such as a number of chemotherapeutic drugs employed for treatment of patients with different types of cancer, are able to ignite antitumor immune responses through the upregulation of immunostimulatory stress-regulated molecules (e.g. NKG2D ligands), the same agents can also increase the expression of immunosuppressive axis, including certain inhibitory immune checkpoints, hence favouring cancer immunoevasion. Consequently, there is an urgent call for unravelling the precise impact of drugs approved for cancer management that rely on cell stress responses -and the type of cell stress modulated by these compounds- on cancer immunosurveillance and immunotherapeutics.

## Abbreviatons:

ATM: – ataxia-telangiectasia mutated,
CML: – chronic myelogenous leukemia,
CRT: – calreticulin,
DC: – dendritic cell,
DDR: – DNA damage response,
ER: – endoplasmic reticulum,
GZMB: – granzyme B,
HIF: – hypoxia-inducible factor,
HRE: – hypoxia response element,
HSF: – heat shock transcription factor,
HSP: – heat shock protein,
ICB: – immune checkpoint blockade,
IFNγ: – interferon γ,
LLC: – Lewis lung carcinoma,
MDSC: – myeloid-derived suppressor cell,
MHC: – major histocompatibility complex,
MICA: – MHC class I polypeptide-related sequence,
MM: – multiple myeloma,
mTOR: – mammalian target of rapamycin,
NK: – natural killer,
ROS: – reactive oxygen species,
TAM: – tumor-associated macrophage,
TME: – tumor microenvironment,
Treg: – regulatory T cell,
UPR: – unfolded protein response,
VEGF: – vascular endothelial growth factor.

## References

[1] Barnes TA, Amir E (2017). HYPE or HOPE: the prognostic value of infiltrating immune cells in cancer.. Br J Cancer.

[2] Wherry EJ, Kurachi M (2015). Molecular and cellular insights into T cell exhaustion.. Nat Rev Immunol.

[3] Park YJ, Kuen DS, Chung Y (2018). Future prospects of immune checkpoint blockade in cancer: from response prediction to overcoming resistance.. Exp Mol Med.

[4] Gonzalez S, Lopez-Soto A, Suarez-Alvarez B, Lopez-Vazquez A, Lopez-Larrea C (2008). NKG2D ligands: key targets of the immune response.. Trends Immunol.

[5] Lopez-Soto A, Huergo-Zapico L, Acebes-Huerta A, Villa-Alvarez M, Gonzalez S (2015). NKG2D signaling in cancer immunosurveillance.. Int J Cancer.

[6] Lopez-Soto A, Gonzalez S, Smyth MJ, Galluzzi L (2017). Control of Metastasis by NK Cells.. Cancer Cell.

[7] Takeuchi H, Tanaka M, Tanaka A, Tsunemi A, Yamamoto H (2016). Predominance of M2-polarized macrophages in bladder cancer affects angiogenesis, tumor grade and invasiveness.. Oncol Lett.

[8] Aras S, Zaidi MR (2017). TAMeless traitors: macrophages in cancer progression and metastasis.. Br J Cancer.

[9] Zhang M, He Y, Sun X, Li Q, Wang W, Zhao A, Di W (2014). A high M1/M2 ratio of tumor-associated macrophages is associated with extended survival in ovarian cancer patients.. J Ovarian Res.

[10] Georgoudaki AM, Prokopec KE, Boura VF, Hellqvist E, Sohn S, Ostling J, Dahan R, Harris RA, Rantalainen M, Klevebring D, Sund M, Brage SE, Fuxe J, Rolny C, Li F, Ravetch JV, Karlsson MC (2016). Reprogramming Tumor-Associated Macrophages by Antibody Targeting Inhibits Cancer Progression and Metastasis.. Cell Rep.

[11] Genard G, Lucas S, Michiels C (2017). Reprogramming of Tumor-Associated Macrophages with Anticancer Therapies: Radiotherapy versus Chemo- and Immunotherapies.. Front Immunol.

[12] Togashi Y, Shitara K, Nishikawa H (2019). Regulatory T cells in cancer immunosuppression - implications for anticancer therapy.. Nat Rev Clin Oncol.

[13] de Visser KE, Eichten A, Coussens LM (2006). Paradoxical roles of the immune system during cancer development.. Nat Rev Cancer.

[14] Hanahan D, Weinberg RA (2011). Hallmarks of cancer: the next generation.. Cell.

[15] Richter K, Haslbeck M, Buchner J (2010). The heat shock response: life on the verge of death.. Mol Cell.

[16] Gomez-Pastor R, Burchfiel ET, Thiele DJ (2018). Regulation of heat shock transcription factors and their roles in physiology and disease.. Nat Rev Mol Cell Biol.

[17] Mendillo ML, Santagata S, Koeva M, Bell GW, Hu R, Tamimi RM, Fraenkel E, Ince TA, Whitesell L, Lindquist S (2012). HSF1 drives a transcriptional program distinct from heat shock to support highly malignant human cancers.. Cell.

[18] Heimberger T, Andrulis M, Riedel S, Stuhmer T, Schraud H, Beilhack A, Bumm T, Bogen B, Einsele H, Bargou RC, Chatterjee M (2013). The heat shock transcription factor 1 as a potential new therapeutic target in multiple myeloma.. Br J Haematol.

[19] Kourtis N, Lazaris C, Hockemeyer K, Balandran JC, Jimenez AR, Mullenders J, Gong Y, Trimarchi T, Bhatt K, Hu H, Shrestha L, Ambesi-Impiombato A, Kelliher M, Paietta E, Chiosis G, Guzman ML, Ferrando AA, Tsirigos A, Aifantis I (2018). Oncogenic hijacking of the stress response machinery in T cell acute lymphoblastic leukemia.. Nat Med.

[20] Santagata S, Hu R, Lin NU, Mendillo ML, Collins LC, Hankinson SE, Schnitt SJ, Whitesell L, Tamimi RM, Lindquist S, Ince TA (2011). High levels of nuclear heat-shock factor 1 (HSF1) are associated with poor prognosis in breast cancer.. Proc Natl Acad Sci U S A.

[21] Engerud H, Tangen IL, Berg A, Kusonmano K, Halle MK, Oyan AM, Kalland KH, Stefansson I, Trovik J, Salvesen HB, Krakstad C (2014). High level of HSF1 associates with aggressive endometrial carcinoma and suggests potential for HSP90 inhibitors.. Br J Cancer.

[22] Fok JHL, Hedayat S, Zhang L, Aronson LI, Mirabella F, Pawlyn C, Bright MD, Wardell CP, Keats JJ, De Billy E, Rye CS, Chessum NEA, Jones K, Morgan GJ, Eccles SA, Workman P, Davies FE (2018). HSF1 Is Essential for Myeloma Cell Survival and A Promising Therapeutic Target.. Clin Cancer Res.

[23] Meng L, Gabai VL, Sherman MY (2010). Heat-shock transcription factor HSF1 has a critical role in human epidermal growth factor receptor-2-induced cellular transformation and tumorigenesis.. Oncogene.

[24] Dai C, Whitesell L, Rogers AB, Lindquist S (2007). Heat shock factor 1 is a powerful multifaceted modifier of carcinogenesis.. Cell.

[25] Min JN, Huang L, Zimonjic DB, Moskophidis D, Mivechi NF (2007). Selective suppression of lymphomas by functional loss of Hsf1 in a p53-deficient mouse model for spontaneous tumors.. Oncogene.

[26] Li D, Yallowitz A, Ozog L, Marchenko N (2014). A gain-of-function mutant p53-HSF1 feed forward circuit governs adaptation of cancer cells to proteotoxic stress.. Cell Death Dis.

[27] Evans SS, Repasky EA, Fisher DT (2015). Fever and the thermal regulation of immunity: the immune system feels the heat.. Nat Rev Immunol.

[28] Ostberg JR, Ertel BR, Lanphere JA (2005). An important role for granulocytes in the thermal regulation of colon tumor growth.. Immunol Invest.

[29] Ostberg JR, Dayanc BE, Yuan M, Oflazoglu E, Repasky EA (2007). Enhancement of natural killer (NK) cell cytotoxicity by fever-range thermal stress is dependent on NKG2D function and is associated with plasma membrane NKG2D clustering and increased expression of MICA on target cells.. J Leukoc Biol.

[30] Kim JY, Son YO, Park SW, Bae JH, Chung JS, Kim HH, Chung BS, Kim SH, Kang CD (2006). Increase of NKG2D ligands and sensitivity to NK cell-mediated cytotoxicity of tumor cells by heat shock and ionizing radiation.. Exp Mol Med.

[31] Venkataraman GM, Suciu D, Groh V, Boss JM, Spies T (2007). Promoter region architecture and transcriptional regulation of the genes for the MHC class I-related chain A and B ligands of NKG2D.. J Immunol.

[32] Schilling D, Kuhnel A, Tetzlaff F, Konrad S, Multhoff G (2015). NZ28-induced inhibition of HSF1, SP1 and NF-kappaB triggers the loss of the natural killer cell-activating ligands MICA/B on human tumor cells.. Cancer Immunol Immunother.

[33] Dayanc BE, Bansal S, Gure AO, Gollnick SO, Repasky EA (2013). Enhanced sensitivity of colon tumour cells to natural killer cell cytotoxicity after mild thermal stress is regulated through HSF1-mediated expression of MICA.. Int J Hyperthermia.

[34] Fionda C, Soriani A, Malgarini G, Iannitto ML, Santoni A, Cippitelli M (2009). Heat shock protein-90 inhibitors increase MHC class I-related chain A and B ligand expression on multiple myeloma cells and their ability to trigger NK cell degranulation.. J Immunol.

[35] Zou J, Guo Y, Guettouche T, Smith DF, Voellmy R (1998). Repression of heat shock transcription factor HSF1 activation by HSP90 (HSP90 complex) that forms a stress-sensitive complex with HSF1.. Cell.

[36] Wells AD, Malkovsky M (2000). Heat shock proteins, tumor immunogenicity and antigen presentation: an integrated view.. Immunol Today.

[37] Srivastava P (2002). Roles of heat-shock proteins in innate and adaptive immunity.. Nat Rev Immunol.

[38] Bendz H, Ruhland SC, Pandya MJ, Hainzl O, Riegelsberger S, Brauchle C, Mayer MP, Buchner J, Issels RD, Noessner E (2007). Human heat shock protein 70 enhances tumor antigen presentation through complex formation and intracellular antigen delivery without innate immune signaling.. J Biol Chem.

[39] Lakshmikanth T, Burke S, Ali TH, Kimpfler S, Ursini F, Ruggeri L, Capanni M, Umansky V, Paschen A, Sucker A, Pende D, Groh V, Biassoni R, Hoglund P, Kato M, Shibuya K, Schadendorf D, Anichini A, Ferrone S, Velardi A, Karre K, Shibuya A, Carbone E, Colucci F (2009). NCRs and DNAM-1 mediate NK cell recognition and lysis of human and mouse melanoma cell lines in vitro and in vivo.. J Clin Invest.

[40] Dodd K, Nance S, Quezada M, Janke L, Morrison JB, Williams RT, Beere HM (2015). Tumor-derived inducible heat-shock protein 70 (HSP70) is an essential component of anti-tumor immunity.. Oncogene.

[41] Gastpar R, Gehrmann M, Bausero MA, Asea A, Gross C, Schroeder JA, Multhoff G (2005). Heat shock protein 70 surface-positive tumor exosomes stimulate migratory and cytolytic activity of natural killer cells.. Cancer Res.

[42] Elsner L, Muppala V, Gehrmann M, Lozano J, Malzahn D, Bickeboller H, Brunner E, Zientkowska M, Herrmann T, Walter L, Alves F, Multhoff G, Dressel R (2007). The heat shock protein HSP70 promotes mouse NK cell activity against tumors that express inducible NKG2D ligands.. J Immunol.

[43] Sies H, Berndt C, Jones DP (2017). Oxidative Stress.. Annu Rev Biochem.

[44] Droge W (2002). Free radicals in the physiological control of cell function.. Physiol Rev.

[45] Klotz LO, Sanchez-Ramos C, Prieto-Arroyo I, Urbanek P, Steinbrenner H, Monsalve M (2015). Redox regulation of FoxO transcription factors.. Redox Biol.

[46] Morgan MJ, Liu ZG (2011). Crosstalk of reactive oxygen species and NF-kappaB signaling.. Cell Res.

[47] Corcoran A, Cotter TG (2013). Redox regulation of protein kinases.. FEBS J.

[48] Lee SR, Yang KS, Kwon J, Lee C, Jeong W, Rhee SG (2002). Reversible inactivation of the tumor suppressor PTEN by H2O2.. J Biol Chem.

[49] Meng TC, Fukada T, Tonks NK (2002). Reversible oxidation and inactivation of protein tyrosine phosphatases in vivo.. Mol Cell.

[50] Chen K, Kirber MT, Xiao H, Yang Y, Keaney JF (2008). Regulation of ROS signal transduction by NADPH oxidase 4 localization.. J Cell Biol.

[51] Elchuri S, Oberley TD, Qi W, Eisenstein RS, Jackson Roberts L, Van Remmen H, Epstein CJ, Huang TT (2005). CuZnSOD deficiency leads to persistent and widespread oxidative damage and hepatocarcinogenesis later in life.. Oncogene.

[52] Xia C, Meng Q, Liu LZ, Rojanasakul Y, Wang XR, Jiang BH (2007). Reactive oxygen species regulate angiogenesis and tumor growth through vascular endothelial growth factor.. Cancer Res.

[53] Ishikawa K, Takenaga K, Akimoto M, Koshikawa N, Yamaguchi A, Imanishi H, Nakada K, Honma Y, Hayashi J (2008). ROS-generating mitochondrial DNA mutations can regulate tumor cell metastasis.. Science.

[54] Maraldi T, Prata C, Caliceti C, Vieceli Dalla Sega F, Zambonin L, Fiorentini D, Hakim G (2010). VEGF-induced ROS generation from NAD(P)H oxidases protects human leukemic cells from apoptosis.. Int J Oncol.

[55] Maj T, Wang W, Crespo J, Zhang H, Wang W, Wei S, Zhao L, Vatan L, Shao I, Szeliga W, Lyssiotis C, Liu JR, Kryczek I, Zou W (2017). Oxidative stress controls regulatory T cell apoptosis and suppressor activity and PD-L1-blockade resistance in tumor.. Nat immunol.

[56] Zhang Y, Choksi S, Chen K, Pobezinskaya Y, Linnoila I, Liu ZG (2013). ROS play a critical role in the differentiation of alternatively activated macrophages and the occurrence of tumor-associated macrophages.. Cell Res.

[57] Corzo CA, Cotter MJ, Cheng P, Cheng F, Kusmartsev S, Sotomayor E, Padhya T, McCaffrey TV, McCaffrey JC, Gabrilovich DI (2009). Mechanism regulating reactive oxygen species in tumor-induced myeloid-derived suppressor cells.. J Immunol.

[58] Kusmartsev S, Nefedova Y, Yoder D, Gabrilovich DI (2004). Antigen-specific inhibition of CD8+ T cell response by immature myeloid cells in cancer is mediated by reactive oxygen species.. J Immunol.

[59] Kusmartsev S, Su Z, Heiser A, Dannull J, Eruslanov E, Kubler H, Yancey D, Dahm P, Vieweg J (2008). Reversal of myeloid cell-mediated immunosuppression in patients with metastatic renal cell carcinoma.. Clin Cancer Res.

[60] Aydin E, Johansson J, Nazir FH, Hellstrand K, Martner A (2017). Role of NOX2-Derived Reactive Oxygen Species in NK Cell-Mediated Control of Murine Melanoma Metastasis.. Cancer Immunol Res.

[61] Romero AI, Thoren FB, Brune M, Hellstrand K (2006). NKp46 and NKG2D receptor expression in NK cells with CD56dim and CD56bright phenotype: regulation by histamine and reactive oxygen species.. Br J Haematol.

[62] Mellqvist UH, Hansson M, Brune M, Dahlgren C, Hermodsson S, Hellstrand K (2000). Natural killer cell dysfunction and apoptosis induced by chronic myelogenous leukemia cells: role of reactive oxygen species and regulation by histamine.. Blood.

[63] Yamamoto K, Fujiyama Y, Andoh A, Bamba T, Okabe H (2001). Oxidative stress increases MICA and MICB gene expression in the human colon carcinoma cell line (CaCo-2).. Biochim Biophys acta.

[64] Wouters BG, Koritzinsky M (2008). Hypoxia signalling through mTOR and the unfolded protein response in cancer.. Nat Rev Cancer.

[65] Majmundar AJ, Wong WJ, Simon MC (2010). Hypoxia-inducible factors and the response to hypoxic stress.. Mol Cell.

[66] Eales KL, Hollinshead KE, Tennant DA (2016). Hypoxia and metabolic adaptation of cancer cells.. Oncogenesis.

[67] Bernhardt S, Tonsing C, Mitra D, Erdem N, Muller-Decker K, Korf U, Kreutz C, Timmer J, Wiemann S (2019). Functional Proteomics of Breast Cancer Metabolism Identifies GLUL as Responder during Hypoxic Adaptation.. J Proteome Res.

[68] Chen L, Endler A, Shibasaki F (2009). Hypoxia and angiogenesis: regulation of hypoxia-inducible factors via novel binding factors.. Exp Mol Med.

[69] Azoitei N, Becher A, Steinestel K, Rouhi A, Diepold K, Genze F, Simmet T, Seufferlein T (2016). PKM2 promotes tumor angiogenesis by regulating HIF-1alpha through NF-kappaB activation.. Mol Cancer.

[70] Gordan JD, Bertout JA, Hu CJ, Diehl JA, Simon MC (2007). HIF-2alpha promotes hypoxic cell proliferation by enhancing c-myc transcriptional activity.. Cancer Cell.

[71] Finger EC, Castellini L, Rankin EB, Vilalta M, Krieg AJ, Jiang D, Banh A, Zundel W, Powell MB, Giaccia AJ (2015). Hypoxic induction of AKAP12 variant 2 shifts PKA-mediated protein phosphorylation to enhance migration and metastasis of melanoma cells.. Proc Natl Acad Sci U S A.

[72] Rankin EB, Giaccia AJ (2016). Hypoxic control of metastasis.. Science.

[73] Choi BJ, Park SA, Lee SY, Cha YN, Surh YJ (2017). Hypoxia induces epithelial-mesenchymal transition in colorectal cancer cells through ubiquitin-specific protease 47-mediated stabilization of Snail: A potential role of Sox9.. Sci Rep.

[74] Noman MZ, Desantis G, Janji B, Hasmim M, Karray S, Dessen P, Bronte V, Chouaib S (2014). PD-L1 is a novel direct target of HIF-1alpha, and its blockade under hypoxia enhanced MDSC-mediated T cell activation.. J Exp Med.

[75] Ruf M, Moch H, Schraml P (2016). PD-L1 expression is regulated by hypoxia inducible factor in clear cell renal cell carcinoma.. Int J Cancer.

[76] Barsoum IB, Smallwood CA, Siemens DR, Graham CH (2014). A mechanism of hypoxia-mediated escape from adaptive immunity in cancer cells.. Cancer Res.

[77] Dahia PL (2014). Pheochromocytoma and paraganglioma pathogenesis: learning from genetic heterogeneity.. Nat Rev Cancer.

[78] Hao HX, Khalimonchuk O, Schraders M, Dephoure N, Bayley JP, Kunst H, Devilee P, Cremers CW, Schiffman JD, Bentz BG, Gygi SP, Winge DR, Kremer H, Rutter J (2009). SDH5, a gene required for flavination of succinate dehydrogenase, is mutated in paraganglioma.. Science.

[79] Pollard PJ, Briere JJ, Alam NA, Barwell J, Barclay E, Wortham NC, Hunt T, Mitchell M, Olpin S, Moat SJ, Hargreaves IP, Heales SJ, Chung YL, Griffiths JR, Dalgleish A, McGrath JA, Gleeson MJ, Hodgson SV, Poulsom R, Rustin P, Tomlinson IP (2005). Accumulation of Krebs cycle intermediates and over-expression of HIF1alpha in tumours which result from germline FH and SDH mutations.. Human Mol Genet.

[80] Pinato DJ, Black JR, Trousil S, Dina RE, Trivedi P, Mauri FA, Sharma R (2017). Programmed cell death ligands expression in phaeochromocytomas and paragangliomas: Relationship with the hypoxic response, immune evasion and malignant behavior.. Oncoimmunology.

[81] Palsson-McDermott EM, Dyck L, Zaslona Z, Menon D, McGettrick AF, Mills KHG, O'Neill LA (2017). Pyruvate Kinase M2 Is Required for the Expression of the Immune Checkpoint PD-L1 in Immune Cells and Tumors.. Front Immunol.

[82] Palazon A, Tyrakis PA, Macias D, Velica P, Rundqvist H, Fitzpatrick S, Vojnovic N, Phan AT, Loman N, Hedenfalk I, Hatschek T, Lovrot J, Foukakis T, Goldrath AW, Bergh J, Johnson RS (2017). An HIF-1alpha/VEGF-A Axis in Cytotoxic T Cells Regulates Tumor Progression.. Cancer Cell.

[83] Leblond MM, Gerault AN, Corroyer-Dulmont A, MacKenzie ET, Petit E, Bernaudin M, Valable S (2016). Hypoxia induces macrophage polarization and re-education toward an M2 phenotype in U87 and U251 glioblastoma models.. Oncoimmunology.

[84] Laoui D, Van Overmeire E, Di Conza G, Aldeni C, Keirsse J, Morias Y, Movahedi K, Houbracken I, Schouppe E, Elkrim Y, Karroum O, Jordan B, Carmeliet P, Gysemans C, De Baetselier P, Mazzone M, Van Ginderachter JA (2014). Tumor hypoxia does not drive differentiation of tumor-associated macrophages but rather fine-tunes the M2-like macrophage population.. Cancer Res.

[85] Doedens AL, Stockmann C, Rubinstein MP, Liao D, Zhang N, DeNardo DG, Coussens LM, Karin M, Goldrath AW, Johnson RS (2010). Macrophage expression of hypoxia-inducible factor-1 alpha suppresses T-cell function and promotes tumor progression.. Cancer Res.

[86] Imtiyaz HZ, Williams EP, Hickey MM, Patel SA, Durham AC, Yuan LJ, Hammond R, Gimotty PA, Keith B, Simon MC (2010). Hypoxia-inducible factor 2alpha regulates macrophage function in mouse models of acute and tumor inflammation.. J Clin Invest.

[87] Wenes M, Shang M, Di Matteo M, Goveia J, Martin-Perez R, Serneels J, Prenen H, Ghesquiere B, Carmeliet P, Mazzone M (2016). Macrophage Metabolism Controls Tumor Blood Vessel Morphogenesis and Metastasis.. Cell Metabo.

[88] Chiu DK, Xu IM, Lai RK, Tse AP, Wei LL, Koh HY, Li LL, Lee D, Lo RC, Wong CM, Ng IO, Wong CC (2016). Hypoxia induces myeloid-derived suppressor cell recruitment to hepatocellular carcinoma through chemokine (C-C motif) ligand 26.. Hepatology.

[89] Chiu DK, Tse AP, Xu IM, Di Cui J, Lai RK, Li LL, Koh HY, Tsang FH, Wei LL, Wong CM, Ng IO, Wong CC (2017). Hypoxia inducible factor HIF-1 promotes myeloid-derived suppressor cells accumulation through ENTPD2/CD39L1 in hepatocellular carcinoma.. Nat Commun.

[90] Facciabene A, Peng X, Hagemann IS, Balint K, Barchetti A, Wang LP, Gimotty PA, Gilks CB, Lal P, Zhang L, Coukos G (2011). Tumour hypoxia promotes tolerance and angiogenesis via CCL28 and T(reg) cells.. Nature.

[91] Hansen W, Hutzler M, Abel S, Alter C, Stockmann C, Kliche S, Albert J, Sparwasser T, Sakaguchi S, Westendorf AM, Schadendorf D, Buer J, Helfrich I (2012). Neuropilin 1 deficiency on CD4+Foxp3+ regulatory T cells impairs mouse melanoma growth.. J Exp Med.

[92] Doedens AL, Phan AT, Stradner MH, Fujimoto JK, Nguyen JV, Yang E, Johnson RS, Goldrath AW (2013). Hypoxia-inducible factors enhance the effector responses of CD8(+) T cells to persistent antigen.. Nat Immunol.

[93] Gropper Y, Feferman T, Shalit T, Salame TM, Porat Z, Shakhar G (2017). Culturing CTLs under Hypoxic Conditions Enhances Their Cytolysis and Improves Their Anti-tumor Function.. Cell Rep.

[94] Siemens DR, Hu N, Sheikhi AK, Chung E, Frederiksen LJ, Pross H, Graham CH (2008). Hypoxia increases tumor cell shedding of MHC class I chain-related molecule: role of nitric oxide.. Cancer Res.

[95] Barsoum IB, Hamilton TK, Li X, Cotechini T, Miles EA, Siemens DR, Graham CH (2011). Hypoxia induces escape from innate immunity in cancer cells via increased expression of ADAM10: role of nitric oxide.. Cancer Res.

[96] Sceneay J, Chow MT, Chen A, Halse HM, Wong CS, Andrews DM, Sloan EK, Parker BS, Bowtell DD, Smyth MJ, Moller A (2012). Primary tumor hypoxia recruits CD11b+/Ly6Cmed/Ly6G+ immune suppressor cells and compromises NK cell cytotoxicity in the premetastatic niche.. Cancer Res.

[97] Baginska J, Viry E, Berchem G, Poli A, Noman MZ, van Moer K, Medves S, Zimmer J, Oudin A, Niclou SP, Bleackley RC, Goping IS, Chouaib S, Janji B (2013). Granzyme B degradation by autophagy decreases tumor cell susceptibility to natural killer-mediated lysis under hypoxia.. Proc Natl Acad Sci U S A.

[98] Berchem G, Noman MZ, Bosseler M, Paggetti J, Baconnais S, Le Cam E, Nanbakhsh A, Moussay E, Mami-Chouaib F, Janji B, Chouaib S (2016). Hypoxic tumor-derived microvesicles negatively regulate NK cell function by a mechanism involving TGF-beta and miR23a transfer.. Oncoimmunology.

[99] Krzywinska E, Kantari-Mimoun C, Kerdiles Y, Sobecki M, Isagawa T, Gotthardt D, Castells M, Haubold J, Millien C, Viel T, Tavitian B, Takeda N, Fandrey J, Vivier E, Sexl V, Stockmann C (2017). Loss of HIF-1alpha in natural killer cells inhibits tumour growth by stimulating non-productive angiogenesis.. Nat Commun.

[100] Hetz C (2012). The unfolded protein response: controlling cell fate decisions under ER stress and beyond.. Nat Rev Mol Cell Biol.

[101] Bobrovnikova-Marjon E, Grigoriadou C, Pytel D, Zhang F, Ye J, Koumenis C, Cavener D, Diehl JA (2010). PERK promotes cancer cell proliferation and tumor growth by limiting oxidative DNA damage.. Oncogene.

[102] Logue SE, McGrath EP, Cleary P, Greene S, Mnich K, Almanza A, Chevet E, Dwyer RM, Oommen A, Legembre P, Godey F, Madden EC, Leuzzi B, Obacz J, Zeng Q, Patterson JB, Jager R, Gorman AM, Samali A (2018). Inhibition of IRE1 RNase activity modulates the tumor cell secretome and enhances response to chemotherapy.. Nat Commun.

[103] Rosati E, Sabatini R, Rampino G, De Falco F, Di Ianni M, Falzetti F, Fettucciari K, Bartoli A, Screpanti I, Marconi P (2010). Novel targets for endoplasmic reticulum stress-induced apoptosis in B-CLL.. Blood.

[104] Galluzzi L, Buque A, Kepp O, Zitvogel L, Kroemer G (2017). Immunogenic cell death in cancer and infectious disease.. Nat Rev Immunol.

[105] Senovilla L, Vitale I, Martins I, Tailler M, Pailleret C, Michaud M, Galluzzi L, Adjemian S, Kepp O, Niso-Santano M, Shen S, Marino G, Criollo A, Boileve A, Job B, Ladoire S, Ghiringhelli F, Sistigu A, Yamazaki T, Rello-Varona S, Locher C, Poirier-Colame V, Talbot M, Valent A, Berardinelli F, Antoccia A, Ciccosanti F, Fimia GM, Piacentini M, Fueyo A (2012). An immunosurveillance mechanism controls cancer cell ploidy.. Science.

[106] Aranda F, Chaba K, Bloy N, Garcia P, Bordenave C, Martins I, Stoll G, Tesniere A, Kroemer G, Senovilla L (2018). Immune effectors responsible for the elimination of hyperploid cancer cells.. Oncoimmunology.

[107] Acebes-Huerta A, Lorenzo-Herrero S, Folgueras AR, Huergo-Zapico L, Lopez-Larrea C, Lopez-Soto A, Gonzalez S (2016). Drug-induced hyperploidy stimulates an antitumor NK cell response mediated by NKG2D and DNAM-1 receptors.. Oncoimmunology.

[108] Lopez-Soto A, Gonzalez S, Lopez-Larrea C, Kroemer G (2017). Immunosurveillance of Malignant Cells with Complex Karyotypes.. Trends Cell Biol.

[109] Cubillos-Ruiz JR, Silberman PC, Rutkowski MR, Chopra S, Perales-Puchalt A, Song M, Zhang S, Bettigole SE, Gupta D, Holcomb K, Ellenson LH, Caputo T, Lee AH, Conejo-Garcia JR, Glimcher LH (2015). ER Stress Sensor XBP1 Controls Anti-tumor Immunity by Disrupting Dendritic Cell Homeostasis.. Cell.

[110] Song M, Sandoval TA, Chae CS, Chopra S, Tan C, Rutkowski MR, Raundhal M, Chaurio RA, Payne KK, Konrad C, Bettigole SE, Shin HR, Crowley MJP, Cerliani JP, Kossenkov AV, Motorykin I, Zhang S, Manfredi G, Zamarin D, Holcomb K, Rodriguez PC, Rabinovich GA, Conejo-Garcia JR, Glimcher LH, Cubillos-Ruiz JR (2018). IRE1alpha-XBP1 controls T cell function in ovarian cancer by regulating mitochondrial activity.. Nature.

[111] Condamine T, Kumar V, Ramachandran IR, Youn JI, Celis E, Finnberg N, El-Deiry WS, Winograd R, Vonderheide RH, English NR, Knight SC, Yagita H, McCaffrey JC, Antonia S, Hockstein N, Witt R, Masters G, Bauer T, Gabrilovich DI (2014). ER stress regulates myeloid-derived suppressor cell fate through TRAIL-R-mediated apoptosis.. J Clin Invest.

[112] Thevenot PT, Sierra RA, Raber PL, Al-Khami AA, Trillo-Tinoco J, Zarreii P, Ochoa AC, Cui Y, Del Valle L, Rodriguez PC (2014). The stress-response sensor chop regulates the function and accumulation of myeloid-derived suppressor cells in tumors.. Immunity.

[113] Mahadevan NR, Rodvold J, Sepulveda H, Rossi S, Drew AF, Zanetti M (2011). Transmission of endoplasmic reticulum stress and pro-inflammation from tumor cells to myeloid cells.. Proc Natl Acad Sci U S A.

[114] Mahadevan NR, Anufreichik V, Rodvold JJ, Chiu KT, Sepulveda H, Zanetti M (2012). Cell-extrinsic effects of tumor ER stress imprint myeloid dendritic cells and impair CD8(+) T cell priming.. PloS One.

[115] Giglia-Mari G, Zotter A, Vermeulen W (2011). DNA damage response.. Cold Spring Harb Perspect Biol.

[116] Tubbs A, Nussenzweig A (2017). Endogenous DNA Damage as a Source of Genomic Instability in Cancer.. Cell.

[117] Bartkova J, Bakkenist CJ, Rajpert-De Meyts E, Skakkebaek NE, Sehested M, Lukas J, Kastan MB, Bartek J (2005). ATM activation in normal human tissues and testicular cancer.. Cell Cycle.

[118] Bartkova J, Horejsi Z, Koed K, Kramer A, Tort F, Zieger K, Guldberg P, Sehested M, Nesland JM, Lukas C, Orntoft T, Lukas J, Bartek J (2005). DNA damage response as a candidate anti-cancer barrier in early human tumorigenesis.. Nature.

[119] Gorgoulis VG, Vassiliou LV, Karakaidos P, Zacharatos P, Kotsinas A, Liloglou T, Venere M, Ditullio RA, Kastrinakis NG, Levy B, Kletsas D, Yoneta A, Herlyn M, Kittas C, Halazonetis TD (2005). Activation of the DNA damage checkpoint and genomic instability in human precancerous lesions.. Nature.

[120] Salgia R, Kulkarni P (2018). The Genetic/Non-genetic Duality of Drug 'Resistance' in Cancer.. Trends Cancer.

[121] Ioannidou A, Goulielmaki E, Garinis GA (2016). DNA Damage: From Chronic Inflammation to Age-Related Deterioration.. Front Genet.

[122] Rooney MS, Shukla SA, Wu CJ, Getz G, Hacohen N (2015). Molecular and genetic properties of tumors associated with local immune cytolytic activity.. Cell.

[123] Van Allen EM, Miao D, Schilling B, Shukla SA, Blank C, Zimmer L, Sucker A, Hillen U, Foppen MHG, Goldinger SM, Utikal J, Hassel JC, Weide B, Kaehler KC, Loquai C, Mohr P, Gutzmer R, Dummer R, Gabriel S, Wu CJ, Schadendorf D, Garraway LA (2015). Genomic correlates of response to CTLA-4 blockade in metastatic melanoma.. Science.

[124] Rizvi NA, Hellmann MD, Snyder A, Kvistborg P, Makarov V, Havel JJ, Lee W, Yuan J, Wong P, Ho TS, Miller ML, Rekhtman N, Moreira AL, Ibrahim F, Bruggeman C, Gasmi B, Zappasodi R, Maeda Y, Sander C, Garon EB, Merghoub T, Wolchok JD, Schumacher TN, Chan TA (2015). Cancer immunology. Mutational landscape determines sensitivity to PD-1 blockade in non-small cell lung cancer.. Science.

[125] Gasser S, Orsulic S, Brown EJ, Raulet DH (2005). The DNA damage pathway regulates innate immune system ligands of the NKG2D receptor.. Nature.

[126] Soriani A, Zingoni A, Cerboni C, Iannitto ML, Ricciardi MR, Di Gialleonardo V, Cippitelli M, Fionda C, Petrucci MT, Guarini A, Foa R, Santoni A (2009). ATM-ATR-dependent up-regulation of DNAM-1 and NKG2D ligands on multiple myeloma cells by therapeutic agents results in enhanced NK-cell susceptibility and is associated with a senescent phenotype.. Blood.

[127] Sato H, Niimi A, Yasuhara T, Permata TBM, Hagiwara Y, Isono M, Nuryadi E, Sekine R, Oike T, Kakoti S, Yoshimoto Y, Held KD, Suzuki Y, Kono K, Miyagawa K, Nakano T, Shibata A (2017). DNA double-strand break repair pathway regulates PD-L1 expression in cancer cells.. Nat Commun.

[128] Shiloh Y, Ziv Y (2013). The ATM protein kinase: regulating the cellular response to genotoxic stress, and more.. Nat Rev Mol Cell Biol.

[129] Textor S, Fiegler N, Arnold A, Porgador A, Hofmann TG, Cerwenka A (2011). Human NK cells are alerted to induction of p53 in cancer cells by upregulation of the NKG2D ligands ULBP1 and ULBP2.. Cancer Res.

[130] Li H, Lakshmikanth T, Garofalo C, Enge M, Spinnler C, Anichini A, Szekely L, Karre K, Carbone E, Selivanova G (2011). Pharmacological activation of p53 triggers anticancer innate immune response through induction of ULBP2.. Cell Cycle.

[131] Soriani A, Iannitto ML, Ricci B, Fionda C, Malgarini G, Morrone S, Peruzzi G, Ricciardi MR, Petrucci MT, Cippitelli M, Santoni A (2014). Reactive oxygen species- and DNA damage response-dependent NK cell activating ligand upregulation occurs at transcriptional levels and requires the transcriptional factor E2F1.. J Immunol.

[132] Li T, Chen ZJ (2018). The cGAS-cGAMP-STING pathway connects DNA damage to inflammation, senescence, and cancer.. J Exp Med.

[133] Woo SR, Fuertes MB, Corrales L, Spranger S, Furdyna MJ, Leung MY, Duggan R, Wang Y, Barber GN, Fitzgerald KA, Alegre ML, Gajewski TF (2014). STING-dependent cytosolic DNA sensing mediates innate immune recognition of immunogenic tumors.. Immunity.

[134] Wang H, Hu S, Chen X, Shi H, Chen C, Sun L, Chen ZJ (2017). cGAS is essential for the antitumor effect of immune checkpoint blockade.. Proc Natl Acad Sci U S A.

[135] Corrales L, Glickman LH, McWhirter SM, Kanne DB, Sivick KE, Katibah GE, Woo SR, Lemmens E, Banda T, Leong JJ, Metchette K, Dubensky TW, Gajewski TF (2015). Direct Activation of STING in the Tumor Microenvironment Leads to Potent and Systemic Tumor Regression and Immunity.. Cell Rep.

[136] Demaria O, De Gassart A, Coso S, Gestermann N, Di Domizio J, Flatz L, Gaide O, Michielin O, Hwu P, Petrova TV, Martinon F, Modlin RL, Speiser DE, Gilliet M (2015). STING activation of tumor endothelial cells initiates spontaneous and therapeutic antitumor immunity.. Proc Natl Acad Sci U S A.

[137] Ager CR, Reilley MJ, Nicholas C, Bartkowiak T, Jaiswal AR, Curran MA (2017). Intratumoral STING Activation with T-cell Checkpoint Modulation Generates Systemic Antitumor Immunity.. Cancer Immunol Res.

[138] Xu MM, Pu Y, Han D, Shi Y, Cao X, Liang H, Chen X, Li XD, Deng L, Chen ZJ, Weichselbaum RR, Fu YX (2017). Dendritic Cells but Not Macrophages Sense Tumor Mitochondrial DNA for Cross-priming through Signal Regulatory Protein alpha Signaling.. Immunity.

[139] Lam AR, Bert NL, Ho SS, Shen YJ, Tang LF, Xiong GM, Croxford JL, Koo CX, Ishii KJ, Akira S, Raulet DH, Gasser S (2014). RAE1 ligands for the NKG2D receptor are regulated by STING-dependent DNA sensor pathways in lymphoma.. Cancer Res.

[140] Wellen KE, Thompson CB (2010). Cellular metabolic stress: considering how cells respond to nutrient excess.. Mol Cell.

[141] Ward PS, Thompson CB (2012). Metabolic reprogramming: a cancer hallmark even warburg did not anticipate.. Cancer Cell.

[142] Pavlova NN, Thompson CB (2016). The Emerging Hallmarks of Cancer Metabolism.. Cell Metab.

[143] Choi H, Na KJ (2018). Pan-cancer analysis of tumor metabolic landscape associated with genomic alterations.. Mol Cancer.

[144] Kim J, Kim J, Bae JS (2016). ROS homeostasis and metabolism: a critical liaison for cancer therapy.. Exp Mol Med.

[145] Samanta D, Semenza GL (2018). Metabolic adaptation of cancer and immune cells mediated by hypoxia-inducible factors.. Biochim Biophys Acta Rev Cancer.

[146] Levine AJ, Puzio-Kuter AM (2010). The control of the metabolic switch in cancers by oncogenes and tumor suppressor genes.. Science.

[147] Galluzzi L, Kepp O, Vander Heiden MG, Kroemer G (2013). Metabolic targets for cancer therapy.. Nat Rev Drug Discov.

[148] DeBerardinis RJ, Chandel NS (2016). Fundamentals of cancer metabolism.. Sci Adv.

[149] Allison KE, Coomber BL, Bridle BW (2017). Metabolic reprogramming in the tumour microenvironment: a hallmark shared by cancer cells and T lymphocytes.. Immunology.

[150] Bantug GR, Galluzzi L, Kroemer G, Hess C (2018). The spectrum of T cell metabolism in health and disease.. Nat Rev Immunol.

[151] Biswas SK (2015). Metabolic Reprogramming of Immune Cells in Cancer Progression.. Immunity.

[152] Challier J, Bruniquel D, Sewell AK, Laugel B (2013). Adenosine and cAMP signalling skew human dendritic cell differentiation towards a tolerogenic phenotype with defective CD8(+) T-cell priming capacity.. Immunology.

[153] Novitskiy SV, Ryzhov S, Zaynagetdinov R, Goldstein AE, Huang Y, Tikhomirov OY, Blackburn MR, Biaggioni I, Carbone DP, Feoktistov I, Dikov MM (2008). Adenosine receptors in regulation of dendritic cell differentiation and function.. Blood.

[154] Young A, Ngiow SF, Gao Y, Patch AM, Barkauskas DS, Messaoudene M, Lin G, Coudert JD, Stannard KA, Zitvogel L, Degli-Esposti MA, Vivier E, Waddell N, Linden J, Huntington ND, Souza-Fonseca-Guimaraes F, Smyth MJ (2018). A2AR Adenosine Signaling Suppresses Natural Killer Cell Maturation in the Tumor Microenvironment.. Cancer Res.

[155] Priebe T, Platsoucas CD, Nelson JA (1990). Adenosine receptors and modulation of natural killer cell activity by purine nucleosides.. Cancer Res.

[156] Allard B, Pommey S, Smyth MJ, Stagg J (2013). Targeting CD73 enhances the antitumor activity of anti-PD-1 and anti-CTLA-4 mAbs.. Clin Cancer Res.

[157] Ohta A, Kini R, Ohta A, Subramanian M, Madasu M, Sitkovsky M (2012). The development and immunosuppressive functions of CD4(+) CD25(+) FoxP3(+) regulatory T cells are under influence of the adenosine-A2A adenosine receptor pathway.. Front Immunol.

[158] Sevigny CP, Li L, Awad AS, Huang L, McDuffie M, Linden J, Lobo PI, Okusa MD (2007). Activation of adenosine 2A receptors attenuates allograft rejection and alloantigen recognition.. J Immunol.

[159] Leone RD, Sun IM, Oh MH, Sun IH, Wen J, Englert J, Powell JD (2018). Inhibition of the adenosine A2a receptor modulates expression of T cell coinhibitory receptors and improves effector function for enhanced checkpoint blockade and ACT in murine cancer models.. Cancer Immunol Immunother.

[160] Willingham SB, Ho PY, Hotson A, Hill C, Piccione EC, Hsieh J, Liu L, Buggy JJ, McCaffery I, Miller RA (2018). A2AR Antagonism with CPI-444 Induces Antitumor Responses and Augments Efficacy to Anti-PD-(L)1 and Anti-CTLA-4 in Preclinical Models.. Cancer Immunol Res.

[161] Young A, Ngiow SF, Barkauskas DS, Sult E, Hay C, Blake SJ, Huang Q, Liu J, Takeda K, Teng MWL, Sachsenmeier K, Smyth MJ (2016). Co-inhibition of CD73 and A2AR Adenosine Signaling Improves Anti-tumor Immune Responses.. Cancer Cell.

[162] Dietl K, Renner K, Dettmer K, Timischl B, Eberhart K, Dorn C, Hellerbrand C, Kastenberger M, Kunz-Schughart LA, Oefner PJ, Andreesen R, Gottfried E, Kreutz MP (2010). Lactic acid and acidification inhibit TNF secretion and glycolysis of human monocytes.. J Immunol.

[163] Colegio OR, Chu NQ, Szabo AL, Chu T, Rhebergen AM, Jairam V, Cyrus N, Brokowski CE, Eisenbarth SC, Phillips GM, Cline GW, Phillips AJ, Medzhitov R (2014). Functional polarization of tumour-associated macrophages by tumour-derived lactic acid.. Nature.

[164] Netea-Maier RT, Smit JWA, Netea MG (2018). Metabolic changes in tumor cells and tumor-associated macrophages: A mutual relationship.. Cancer Lett.

[165] Patsoukis N, Bardhan K, Chatterjee P, Sari D, Liu B, Bell LN, Karoly ED, Freeman GJ, Petkova V, Seth P, Li L, Boussiotis VA (2015). PD-1 alters T-cell metabolic reprogramming by inhibiting glycolysis and promoting lipolysis and fatty acid oxidation.. Nat Commun.

[166] Palmieri EM, Menga A, Martin-Perez R, Quinto A, Riera-Domingo C, De Tullio G, Hooper DC, Lamers WH, Ghesquiere B, McVicar DW, Guarini A, Mazzone M, Castegna A (2017). Pharmacologic or Genetic Targeting of Glutamine Synthetase Skews Macrophages toward an M1-like Phenotype and Inhibits Tumor Metastasis.. Cell Rep.

[167] Choi J, Stradmann-Bellinghausen B, Yakubov E, Savaskan NE, Regnier-Vigouroux A (2015). Glioblastoma cells induce differential glutamatergic gene expressions in human tumor-associated microglia/macrophages and monocyte-derived macrophages.. Cancer Biol Ther.

[168] Deng R, Wang SM, Yin T, Ye TH, Shen GB, Li L, Zhao JY, Sang YX, Duan XG, Wei YQ (2013). Inhibition of tumor growth and alteration of associated macrophage cell type by an HO-1 inhibitor in breast carcinoma-bearing mice.. Oncol Res.

[169] Nemeth Z, Li M, Csizmadia E, Dome B, Johansson M, Persson JL, Seth P, Otterbein L, Wegiel B (2015). Heme oxygenase-1 in macrophages controls prostate cancer progression.. Oncotarget.

[170] Zhang Z, Liu Q, Che Y, Yuan X, Dai L, Zeng B, Jiao G, Zhang Y, Wu X, Yu Y, Zhang Y, Yang R (2010). Antigen presentation by dendritic cells in tumors is disrupted by altered metabolism that involves pyruvate kinase M2 and its interaction with SOCS3.. Cancer Res.

[171] Assmann N, O'Brien KL, Donnelly RP, Dyck L, Zaiatz-Bittencourt V, Loftus RM, Heinrich P, Oefner PJ, Lynch L, Gardiner CM, Dettmer K, Finlay DK (2017). Srebp-controlled glucose metabolism is essential for NK cell functional responses.. Nat Immunol.

[172] Donnelly RP, Loftus RM, Keating SE, Liou KT, Biron CA, Gardiner CM, Finlay DK (2014). mTORC1-dependent metabolic reprogramming is a prerequisite for NK cell effector function.. J Immunol.

[173] Keating SE, Zaiatz-Bittencourt V, Loftus RM, Keane C, Brennan K, Finlay DK, Gardiner CM (2016). Metabolic Reprogramming Supports IFN-gamma Production by CD56bright NK Cells.. J Immunol.

[174] Cong J, Wang X, Zheng X, Wang D, Fu B, Sun R, Tian Z, Wei H (2018). Dysfunction of Natural Killer Cells by FBP1-Induced Inhibition of Glycolysis during Lung Cancer Progression.. Cell Metab.

[175] Grohmann U, Bronte V (2010). Control of immune response by amino acid metabolism.. Immunol Rev.

[176] Mondanelli G, Bianchi R, Pallotta MT, Orabona C, Albini E, Iacono A, Belladonna ML, Vacca C, Fallarino F, Macchiarulo A, Ugel S, Bronte V, Gevi F, Zolla L, Verhaar A, Peppelenbosch M, Mazza EMC, Bicciato S, Laouar Y, Santambrogio L, Puccetti P, Volpi C, Grohmann U (2017). A Relay Pathway between Arginine and Tryptophan Metabolism Confers Immunosuppressive Properties on Dendritic Cells.. Immunity.

[177] Narita Y, Kitamura H, Wakita D, Sumida K, Masuko K, Terada S, Nakano K, Nishimura T (2013). The key role of IL-6-arginase cascade for inducing dendritic cell-dependent CD4(+) T cell dysfunction in tumor-bearing mice.. J Immunol.

[178] Norian LA, Rodriguez PC, O'Mara LA, Zabaleta J, Ochoa AC, Cella M, Allen PM (2009). Tumor-infiltrating regulatory dendritic cells inhibit CD8+ T cell function via L-arginine metabolism.. Cancer Res.

[179] Gorgun GT, Whitehill G, Anderson JL, Hideshima T, Maguire C, Laubach J, Raje N, Munshi NC, Richardson PG, Anderson KC (2013). Tumor-promoting immune-suppressive myeloid-derived suppressor cells in the multiple myeloma microenvironment in humans.. Blood.

[180] Khaled YS, Ammori BJ, Elkord E (2014). Increased levels of granulocytic myeloid-derived suppressor cells in peripheral blood and tumour tissue of pancreatic cancer patients.. J Immunol Res.

[181] Ouzounova M, Lee E, Piranlioglu R, El Andaloussi A, Kolhe R, Demirci MF, Marasco D, Asm I, Chadli A, Hassan KA, Thangaraju M, Zhou G, Arbab AS, Cowell JK, Korkaya H (2017). Monocytic and granulocytic myeloid derived suppressor cells differentially regulate spatiotemporal tumour plasticity during metastatic cascade.. Nat Commun.

[182] Yu J, Du W, Yan F, Wang Y, Li H, Cao S, Yu W, Shen C, Liu J, Ren X (2013). Myeloid-derived suppressor cells suppress antitumor immune responses through IDO expression and correlate with lymph node metastasis in patients with breast cancer.. J Immunol.

[183] Fallarino F, Grohmann U, You S, McGrath BC, Cavener DR, Vacca C, Orabona C, Bianchi R, Belladonna ML, Volpi C, Santamaria P, Fioretti MC, Puccetti P (2006). The combined effects of tryptophan starvation and tryptophan catabolites down-regulate T cell receptor zeta-chain and induce a regulatory phenotype in naive T cells.. J Immunol.

[184] Rodriguez PC, Zea AH, DeSalvo J, Culotta KS, Zabaleta J, Quiceno DG, Ochoa JB, Ochoa AC (2003). L-arginine consumption by macrophages modulates the expression of CD3 zeta chain in T lymphocytes.. J Immunol.

[185] Zea AH, Rodriguez PC, Culotta KS, Hernandez CP, DeSalvo J, Ochoa JB, Park HJ, Zabaleta J, Ochoa AC (2004). L-Arginine modulates CD3zeta expression and T cell function in activated human T lymphocytes.. Cell Immunol.

[186] Geiger R, Rieckmann JC, Wolf T, Basso C, Feng Y, Fuhrer T, Kogadeeva M, Picotti P, Meissner F, Mann M, Zamboni N, Sallusto F, Lanzavecchia A (2016). L-Arginine Modulates T Cell Metabolism and Enhances Survival and Anti-tumor Activity.. Cell.

[187] Rodriguez PC, Quiceno DG, Ochoa AC (2007). L-arginine availability regulates T-lymphocyte cell-cycle progression.. Blood.

[188] Lamas B, Vergnaud-Gauduchon J, Goncalves-Mendes N, Perche O, Rossary A, Vasson MP, Farges MC (2012). Altered functions of natural killer cells in response to L-Arginine availability.. Cell Immunol.

[189] Brochez L, Chevolet I, Kruse V (2017). The rationale of indoleamine 2,3-dioxygenase inhibition for cancer therapy.. Eur J Cancer.

[190] Prendergast GC, Malachowski WP, DuHadaway JB, Muller AJ (2017). Discovery of IDO1 Inhibitors: From Bench to Bedside.. Cancer Res.

